# Dietary patterns and hypertension in Chinese adults: a systematic review and meta-analysis

**DOI:** 10.3389/fpubh.2025.1539359

**Published:** 2025-05-07

**Authors:** Xi Wu, Jianwei Liu, Zhuofeng Wang, Shangya Chen, Jiazi Ma, Mao Cao, Yong Yang, Guangjian Wu, Wentao Li, Zhongjun Du

**Affiliations:** ^1^Shandong Academy of Occupational Health and Occupational Medicine, Shandong First Medical University & Shandong Academy of Medical Sciences, Jinan, China; ^2^Shandong Center for Disease Control and Prevention, Jinan, China; ^3^Harbin Second Hospital, Harbin, China

**Keywords:** dietary pattern, hypertension, meta-analysis, Chinese adults, prevalence

## Abstract

**Objective:**

Numerous studies have explored the correlation between dietary patterns and the risk of hypertension, yet the findings have remained indeterminate. We performed a meta-analysis to evaluate how various dietary patterns relate to hypertension risk in the Chinese population.

**Methods:**

Relevant articles published from 1 January 2004 to 14 March 2024 in five databases (PubMed, Scopus, Web of Science, CNKI, and VIP) were searched. Fixed or random-effects models were employed to estimate the multivariable-adjusted odds ratios (ORs) and 95% confidence intervals (CIs) comparing the highest and lowest categories of dietary patterns.

**Results:**

In total, 22 articles were incorporated into the meta-analysis. The pooled results indicated a reduced likelihood for hypertension in the highest compared to the lowest category of the traditional southern Chinese pattern (OR = 0.95, 95% CI: 0.92–0.97, *p* < 0.001). In addition, compared to the lowest category of fruit and dairy pattern, the risk of the highest category had a 25% reduction in hypertension risk (OR = 0.75, 95% CI: 0.64–0.89, *p* = 0.001). Nevertheless, there was no significant correlation between the animal food pattern and the odds of hypertension (OR = 1.06, 95% CI: 0.98–1.15, *p* = 0.171).

**Conclusion:**

The traditional southern Chinese pattern as well as the fruit and dairy pattern was a protective factor for hypertension. High-quality, large-scale studies are needed to confirm the findings of the current meta-analysis further.

## 1 Introduction

Hypertension (HTN) is one of the most prevalent chronic diseases and one of the principal causes of public health issues, leading to a huge healthcare burden worldwide (1). Cardiovascular disease, chronic kidney disease, and cognitive impairment are common complications of HTN (2–4). According to the World Health Organization, approximately 1.28 billion adults aged 30–79 years worldwide are afflicted with HTN in 2023, with less than half (42%) receiving a diagnosis and treatment (5). The prevalence and overall burden of HTN are increasing on a global scale, particularly in low- and middle-income nations (6). Data from the China Chronic Disease and Risk Factor Surveillance indicates that 27.5% of Chinese adults are affected by HTN, with low rates of awareness and treatment among those diagnosed (7).

It is widely recognized that HTN is subject to a combination of genetic susceptibility and environmental factors (8). Diverse non-pharmacological strategies have been proposed to prevent and control HTN, such as weight reduction, healthy eating patterns, moderate alcohol intake, exercise enhancement, dietary sodium restriction and potassium supplementation (9). Numerous studies have shown that the intake of individual nutrients or foods, such as sodium (10), lycopene (11), and soy (12), are strongly associated with lower blood pressure. However, there are interactions among different foods or nutrients, and a single evaluation of the health effect of nutrients or a specific food cannot adequately and realistically reflect the comprehensive impact of the overall dietary situation on human health (13). Therefore, dietary pattern, which is a combination of multiple foods, is regarded as a more accurate indicator of overall food consumption and nutritional condition. In fact, the Dietary Approaches to Stop Hypertension (DASH) diet has been demonstrated to be efficacious in reducing the risk of HTN and has been proposed for HTN management (14).

As a result of rapid economic development, the dietary pattern of Chinese adults has changed significantly, as evidenced by a decrease in the consumption of vegetables and cereals, an increase in the consumption of animal foods (of which pork dominates), and a slight increase in the consumption of eggs, fish, and dairy products (15). In recent years, scholars in China have paid increasing attention to the correlation between dietary patterns and the likelihood of HTN (16–19). Nevertheless, the findings from these studies have shown a lack of consistency. Therefore, we carried out a revised meta-analysis of observational studies to provide epidemiological evidence on how dietary patterns relate to the risk of HTN.

## 2 Materials and methods

### 2.1 Searching strategy

This article followed the PRISMA Standard guidelines (20). An exhaustive literature retrieval was undertaken through PubMed, Scopus, Web of Science, CNKI, and VIP database from 1 January 2004 to 14 March 2024. The subsequent key words and medical subject headings were implemented during the search: (diet pattern OR dietary pattern OR dietary patterns OR food pattern OR food patterns OR eating pattern OR eating patterns) AND (hypertension OR high blood pressure OR high blood pressures) AND (adults OR adult) AND (Chinese OR China). What’s more, we conducted a comprehensive evaluation of the reference lists of pertinent studies in order to identify additional studies.

### 2.2 Eligibility criteria

Articles that were in the original report and met the criteria in Table 1 were eligible for inclusion the meta-analysis. To minimize error, only the dietary patterns with similar factor loadings of foods were selected.

**Table 1:** Inclusion and exclusion criteria for selection.

	Include	Exclude
Participants	Chinese adults ≥ 18 years	Pregnant or lactating women Reside abroad
Exposure	Dietary patterns that described the characteristics of the overall diet Dietary patterns derived from factor analysis and/or principal component analysis	The intake of specific nutrients or foods
Outcome	Hypertension (defined by combining SBP > 140 mmHg and/or DBP > 90 mmHg, a self-reported diagnosis of hypertension, or by taking anti-hypertensive medication)	Blood pressure
Study design	Cross-sectional and longitudinal studies	Intervention studies
Other aspects	Report the RRs, HRs, or ORs and the corresponding 95% CI for the highest compared with the lowest category of dietary patterns Adjusted for confounding factors	Explore the relationship of dietary pattern scores (continuous variable) with SBP and/or DBP Pooled analysis, academic conferences, *in vitro*, and/or animal studies, reviews, systematic reviews, meta-analyses, case studies, guidelines and commentaries Duplicate published, unpublished, and ongoing studies Lack detailed data

SBP, systolic blood pressure. DBP, diastolic blood pressure. RRs, relative risks. HRs, hazards ratios. ORs, odds ratios.

### 2.3 Data extraction

Three researchers autonomously reviewed the literature, gathered the data, and verified it for accuracy. In case of discrepancies, a consensus should be reached through consultation with a fourth party. The subsequent information was extracted for each specified study: first author’s surname, publication year, study design, number of participants, characteristics of population, diet assessment method, dietary patterns identified and potential confounders adjusted in analysis. With respect to multiple estimates, those with adjustment for the most confounding factors were chosen.

### 2.4 Quality assessment

The quality of the identified studies were evaluated using the National Institutes of Health Quality Assessment Tool for Observational Cohort and Cross-Sectional Studies (21). The evaluation tool comprises 14 criteria of equal weight, resulting in a maximum of fourteen points being assigned to each study. A score below 7 implied a high risk of bias, while a score between 7 and 10 suggested a moderate risk, and a score of 11–14 was deemed as low risk.

### 2.5 Statistical analysis

To assess HTN risk in the highest compared to the lowest category of the traditional southern Chinese pattern, the fruit and dairy pattern and the animal food pattern, we implemented this meta-analysis. The I^2^ and Q statistic were employed to determine statistical heterogeneity (22). I^2^ ≤ 50% and *p* > 0.10 suggested no heterogeneity among studies, and a fixed-effects model (Mantel–Haenszel method) was employed. Conversely, a random-effects model (DerSimonian and Laird method) was utilized (23). Multivariable adjusted odds ratios (ORs) with 95% confidence intervals (CIs) from each study were weighted and combined to calculate the summary OR and its 95% CI. We further carried out subgroup analysis based on study design (cross-sectional and cohort), sample size (≥ 5,000 and < 5,000), and with or without adjustment for several confounders to assess whether the differences affected our study conclusions. The robustness of the results was demonstrated by leave-one-out sensitivity analysis. Egger’s test, Begg’s test, and funnel plots were implemented to evaluate publication bias (24, 25). If substantial publication bias was identified, the trim-and-fill method was implemented to mitigate it (26). All statistical analyses were conducted with STATA version 17.0 (StataCorp LLC, College Station, TX 77845, USA). All *p* values were two-tailed, and *p* values < 0.05 were deemed as statistically significant, except where otherwise specified.

## 3 Results

### 3.1 Study selection

**Figure 1:**
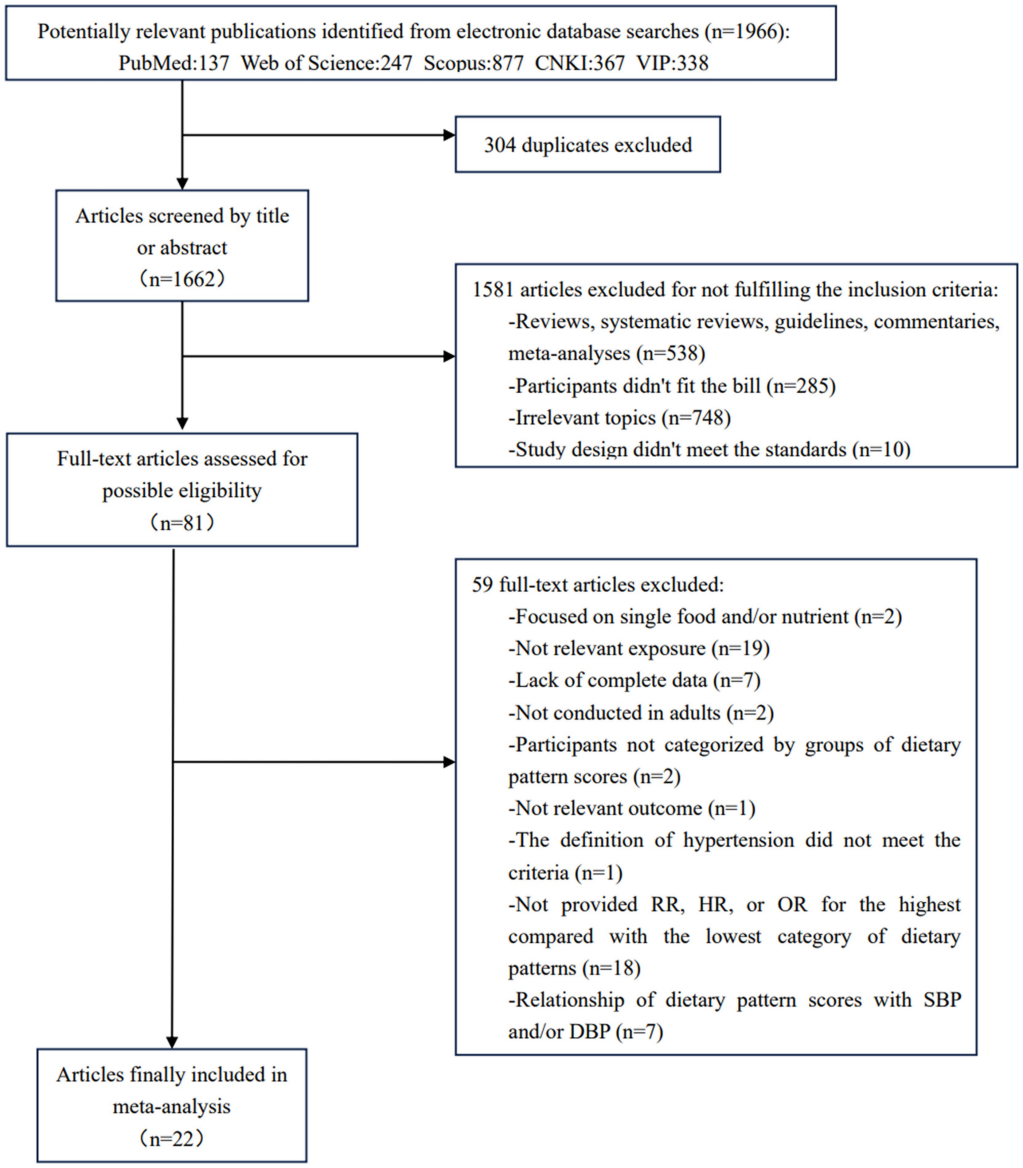
Flow chart of study selection. PubMed, Public Medicine. CNKI, China national knowledge infrastructure. VIP, VIP Database for Chinese Technical Periodicals. RR, relative risk. HR, hazard ratio. OR, odds ratio. SBP, systolic blood pressure. DBP, diastolic blood pressure.

After a primary search, we screened 1,966 articles in total, of which 304 duplicates were deleted. Then, we eliminated 1,581 articles by going through the titles and abstracts whose study population, topic, or outcomes were deemed irrelevant. We excluded 59 articles after performing a thorough review of the remaining 81. Ultimately, 22 articles were eligible for inclusion in this meta-analysis. The flowchart of the stu2dy selection procedure is illustrated in Figure 1.

### 3.2 Study characteristics

The features of the identified 22 articles (27–48) are depicted in Table 2. Among them, one article (40) reported the results of two cross-sectional studies. Another article (31) conducted both a cross-sectional study and a cohort study. Consequently, the present meta-analysis comprised 20 cross-sectional studies (27–29, 31–46) and 4 longitudinal studies (30, 31, 47, 48). The papers were released from 2011 to 2023. Of these, five articles (27, 28, 31, 32, 42) analyzed the relationship between HTN and dietary patterns in gender-specific populations, and the remaining 17 articles (29, 30, 33–41, 43–48) examined the general population. The sample sizes of these investigations varied from 426 to 53,241. Dietary data were collected using a 3-day 24-h dietary review in 9 articles (27–31, 45–48), a food frequency questionnaire in 11 articles (32–36, 38, 40, 42–45), and a semi-quantitative food frequency questionnaire in 3 articles (37, 39, 41).

**Table 2:** Main characteristics of studies included.

Author Publication Year	Study design	Participants	Population	Diet assessment method	Dietary patterns identified	Factors adjusted for in analysis
Liu (27)	Cross-sectional	2,596 (43.7% males)	Adults aged 18–75 years in the CHNS between 1997 and 2009	3 day 24 h recall	Men: common, healthy, snack-fast-food, meat, alcohol Women: sober, fruits and milk, meat, common	UI, age, household income, education, occupation, alcohol drinking, smoking, WPA, total intake of energy, BMI
Cheng (2006–2009) (31)	Cohort	4,493 (45.3% males)	Adults aged 18–75 years from the CHNS between 2006 and 2009	3 day 24 h recall	Men: traditional southern, western, animal protein Women: traditional southern, western, high-energy high-sodium vegetarian	UI, age, household income, education, alcohol drinking, smoking, physical activity, energy intake, territory
Cheng (2009) (31)	Cross-sectional	7,002 (47.1% males)	Adults aged 18–75 years in the 2009 CHNS	3 day 24 h recall	Traditional southern, western, high-energy high-sodium vegetarian	UI, age, household income, education, alcohol drinking, smoking, WPA, energy intake, territory
Liu et al. (29)	Cross-sectional	1,193 (40.7% males)	Uygur adults in Kashi of Xinjiang	3 day 24 h recall	Traditional, western, meat eating, Uygur specific	Age, sex, BMI
Wang (32)	Cross-sectional	2,508 (34.0% males)	Adults aged ≥ 18 years in Xiamen city, Fujian province	FFQ	Men: condiment, milk and fruit, Minnan characteristic, vegetable and meat Women: high sugar high fat, Minnan characteristic, condiment, fruit and milk	Age, marital status, education level
Xiong et al. (36)	Cross-sectional	2,995 (50.0% males)	Adults aged 30–79 years in Rongchang from National Key R&D Program of China(The China Multi-Ethnic Cohort Study)	FFQ	Dairy fruit, poultry red meat, tuber pickles	Sex, age, marital status, occupation, education, annual household income, physical activity intensity, sedentary time, smoking, alcohol drinking, BMI, diabetes, hyperlipemia, coronary heart disease
Lin (34)	Cross-sectional	1,479 (48.3% males)	Adults aged 35–74 years in Hainan province	FFQ	Traditional food diversity, modern food diversity, modern simplified	Age, education level, central obesity, occupation, BMI, annual household income, alcohol drinking
Tong et al. (35)	Cross-sectional	612 (37.6% males)	Adults aged 35–80 years in Haidian district, Beijing	FFQ	Traditional; animal meat, fish and shrimp; vegetable and milk; processed or preserved food	Sex, age, marital status, education, monthly household income, smoking, alcohol drinking, BMI
He et al. (28)	Cross-sectional	2,196 (47.0% males)	Adults aged over 18 years in the fifth CNNHS between 2010 and 2012	3 day 24 h recall	Refined grains and vegetables, dairy and eggs, organ meat and poultry, coarse grains and beans	Age, occupation, living area, BMI
Xu et al. (30)	Cohort	6,348 (47.3% males)	Adults aged over 60 years in the CHNS between 2004 and 2011	3 day 24 h recall	Traditional, modern	Age, urbanization, gender, marital status, work status, education level, smoking, physical activity, energy, salt, BMI, WC
Hu et al. (39)	Cross-sectional	8,144 (48.6% males)	Adults aged ≥ 18 years in Xi’an city	SQFFQ	Animal foods, beverage, vegetarian	Age, sex, place of residence, education level, marital status, occupational status, physical activity, BMI, WC, complication, smoking, alcohol drinking, sedentary behavior
Wang (45)	Cross-sectional	1,287 (48.1% males)	Adults aged ≥ 18 years in Gusu district, Suzhou city, Jiangsu province	FFQ, 3 day 24 h recall	Vegetables and red meat, pickled products and beverage, fruit and dairy, white meat and dessert	Sex, age, marital status, education, smoking, alcohol drinking, sleeping disorders, BMI, sedentary time, sleep time, diabetes, hyperlipemia, coronary heart disease, cerebral apoplexy and other chronic diseases, energy intake
Li (2010–2012) (40)	Cross-sectional	27,514 (not clear)	2010–2012 CNNHS participants aged ≥ 18 years	FFQ	Legume-vegetable, dairy-snacks, animal foods, main-food	Age, sex, area, physical activity, smoking, alcohol drinking
Li (2015) (40)	Cross-sectional	53,241 (not clear)	Adults aged over 18 years in the 2015 China Choronic Diseases and Nutrition Survey	FFQ	Legume-vegetable, dairy-snacks, animal foods, main-food	Age, sex, physical activity, smoking, alcohol drinking
Ren et al. (42)	Cross-sectional	5,649 (47.2% males)	Adults aged ≥ 18 years in Shanxi province	FFQ	Men: high protein; high fat and sweet; grain, potato and pickles; vegetable and fruit Women: low carbohydrate, grain and vegetable, high protein, high fat and sweet, potato pickles	Age, sex, BMI, smoking, alcohol drinking, marital status, education level, place of residence, daily exercise time, occupation
Liu et al. (44)	Cross-sectional	909 (38.0% males)	Adults aged 35–75 years in the “China project for early screening and comprehensive intervention of cardiovascular diseases”	FFQ	Modern healthy, animal protein, grain	Age, sex, education level, marital status, annual household income, exercise, smoking, alcohol drinking, central obesity, BMI, dyslipidemia, diabetes
Jiang et al. (38)	Cross-sectional	2,654 (46.9% males)	Adults aged ≥ 18 years in the 2015 CHNS	FFQ	Grain, pork and vegetable; egg, milk and soy bean; animal food; alcoholic and aquatic product	Age, gender, area
Cao et al. (41)	Cross-sectional	426 (100% females)	Women aged 18–70 years in Xicheng district, Beijing	SQFFQ	Traditional; meat; fruit, egg and dairy; grain, alcohol and beverage	Age, nationality, education level, income, exercise, smoking
Zhang et al. (47)	Cohort	15,929 (not clear)	Adults aged over 18 years from the CHNS between 1991 and 2018	3 day 24 h recall	Southern, modern, meat	Age, gender, living area, individual income, education level, physical activity, smoking status, alcohol consumption, baseline SBP, baseline DBP, total energy intake, sodium intake, BMI
Zhao et al. (48)	Cohort	3,892 (45.1% males)	Adults aged 18–60 years from the CHNS between 2004 and 2009	3 day 24 h recall	Wheat and dairy, meat, modern, traditional southern, snack000000	Sex, age, residence, region, marital status, education level, per capita annual family income, smoking status, drinking habits, sleep duration, physical activity time, sedentary time, total energy, BMI, SBP at baseline, DBP at baseline
Li et al. (43)	Cross-sectional	1,136 (100% males)	Males aged over 65 years in Sichuan province	FFQ	Animal-based and processed food, traditional food, ovo-lacto vegetarian food,	Age, living status, education level, smoking status, alcohol consumption, total energy intake
Wang et al. (46)	Cross-sectional	2,718 (47.4% males)	Adults aged ≥ 18 years in Suzhou, Jiangsu province	3 day 24 h recall	Rice-vegetable, fast food, fruit-dairy, wheat-meat	Sex, age, energy intake, education, smoking, alcohol drinking, sleeping disorders, daily salt intake, sedentary time, WC, family history of hypertension
Shu et al. (37)	Cross-sectional	860 (30.7% males)	Adults aged 45–76 years in Bengbu, Anhui province	SQFFQ	Rice and vegetables, animal food, fruits and milk, drinking	Sex, age, education, physical activity, BMI
Zhang et al. (33)	Cross-sectional	3,315 (43.5% males)	Permanent residents aged 18–79 in Mentougou district, Beijing	FFQ	Healthy, westernized, traditional	Age, sex, occupation, personal annual income, education, physical activity, smoking, alcohol drinking, BMI, FPG, TC, TG, HDL-C, LDL-C

CHNS, China Health and Nutrition Survey; UI, urbanization index; WPA, work-related physical activity; BMI, body mass index; CNNHS, Chinese Nationwide Nutrition and Health Survey; SQFFQ, semi-quantitative food frequency questionnaire; FFQ, food frequency questionnaire; WC, waist circumference; SBP, systolic blood pressure; DBP, diastolic blood pressure; FPG, fasting plasma glucose; TC, total cholesterol; TG, triglyceride; HDL-C, high-density lipoprotein cholesterol; LDL-C, low-density lipoprotein cholesterol.

### 3.3 Quality assessment

All four of the included cohort studies were found to have high methodological quality (Table 3). By contrast, the remaining 20 cross-sectional studies were classified as having a medium risk of bias. The absence of temporal separation between exposures and outcomes was the main cause of bias risk in the cross-sectional researches.

**Table 3:** Quality assessment of included studies.

Criteria	Liu (27)	Cheng (2006–2009) (31)	Cheng (2009) (31)	Liu et al. (29)	Wang (32)	Xiong et al. (36)	Lin (34)	Tong et al. (35)	He et al. (28)	Xu et al. (30)	Hu et al. (39)	Wang (45)	Li (2010–2012) (40)	Li (2015) (40)	Ren et al. (42)	Liu et al. (44)	Jiang et al. (38)	Cao et al. (41)	Zhang et al. (47)	Zhao et al. (48)	Li et al. (43)	Wang et al. (46)	Shu et al. (37)	Zhang et al. (33)
1. Was the research question or objective in this paper clearly stated?	1	1	1	1	1	1	1	1	1	1	1	1	1	1	1	1	1	1	1	1	1	1	1	1
2. Was the study population clearly specified and defined?	1	1	1	1	1	1	1	1	1	1	1	1	1	1	1	1	1	1	1	1	1	1	1	1
3. Was the participation rate of eligible persons at least 50%?	1	1	1	1	1	1	1	1	1	1	1	1	1	1	1	1	1	1	1	1	1	1	1	1
4. Were all the subjects selected or recruited from the same or similar populations (including the same time period)? Were inclusion and exclusion criteria for being in the study prespecified and applied uniformly to all participants?	1	1	1	1	1	1	1	1	1	1	1	1	1	1	1	1	1	1	1	1	1	1	1	1
5. Was a sample size justification, power description, or variance and effect estimates provided?	1	1	1	1	1	1	1	0	1	1	1	1	1	1	1	1	1	1	1	1	1	1	1	1
6. For the analyses in this paper, were the exposure(s) of interest measured prior to the outcome(s) being measured?	0	1	0	0	0	0	0	0	0	1	0	0	0	0	0	0	0	0	1	1	0	0	0	0
7. Was the timeframe sufficient so that one could reasonably expect to see an association between exposure and outcome if it existed?	0	1	0	0	0	0	0	0	0	1	0	0	0	0	0	0	0	0	1	1	0	0	0	0
8. For exposures that can vary in amount or level, did the study examine different levels of the exposure as related to the outcome (e.g., categories of exposure, or exposure measured as continuous variable)?	1	1	1	1	1	1	1	1	1	1	1	1	1	1	1	1	1	1	1	1	1	1	1	1
9. Were the exposure measures (independent variables) clearly defined, valid, reliable, and implemented consistently across all study participants?	1	1	1	1	1	1	1	1	1	1	1	1	1	1	1	1	1	1	1	1	1	1	1	1
10. Was the exposure(s) assessed more than once over time?	0	1	0	0	0	0	0	0	0	1	0	0	0	0	0	0	0	0	1	1	0	0	0	0
11. Were the outcome measures (dependent variables) clearly defined, valid, reliable, and implemented consistently across all study participants?	1	1	1	1	1	1	1	1	1	1	1	1	1	1	1	1	1	1	1	1	1	1	1	1
12. Were the outcome assessors blinded to the exposure status of participants?	1	1	1	1	1	1	1	1	1	1	1	1	1	1	1	1	1	1	1	1	1	1	1	1
13. Was loss to follow-up after baseline 20% or less?	0	1	0	0	0	0	0	0	0	1	0	0	0	0	0	0	0	0	1	1	0	0	0	0
14. Were key potential confounding variables measured and adjusted statistically for their impact on the relationship between exposure(s) and outcome(s)?	1	1	1	1	1	1	1	1	1	1	1	1	1	1	1	1	1	1	1	1	1	1	1	1
Total score	10	14	10	10	10	10	10	9	10	14	10	10	10	10	10	10	10	10	14	14	10	10	10	10
Risk	M	L	M	M	M	M	M	M	M	L	M	M	M	M	M	M	M	M	L	L	M	M	M	M

L, Low. M, Medium.

### 3.4 Traditional southern Chinese pattern and hypertension

**Figure 2:**
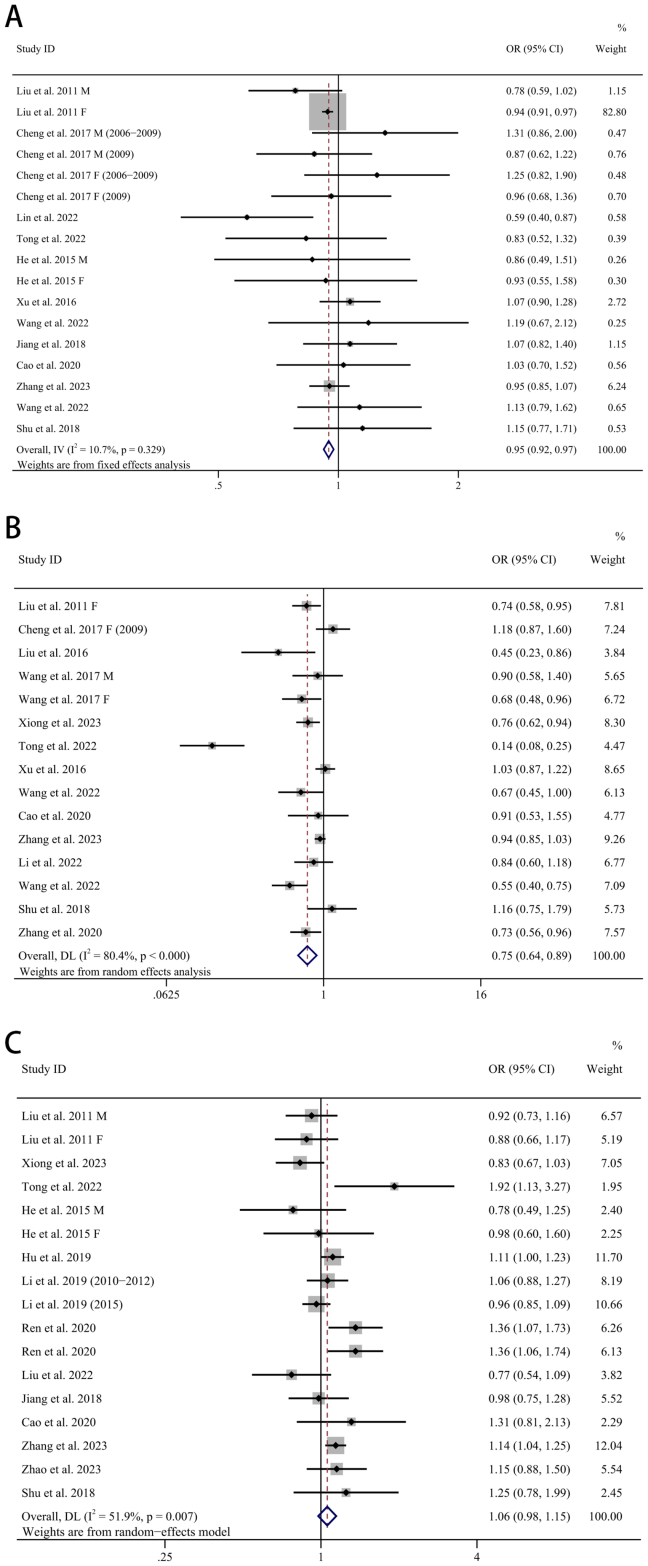
Forest plots of dietary patterns and hypertension. **(A)** Chinese traditional southern dietary pattern and hypertension, **(B)** fruit and dairy dietary pattern and hypertension, **(C)** animal food dietary pattern and hypertension. OR, odds ratio. CI, confidence interval. M, male. F, female.

Of the 24 studies, 13 identified the ‘traditional southern Chinese diet’ as a dietary pattern; 3 of them were longitudinal studies (30, 31, 47) and the remaining 10 were cross-sectional studies (27, 28, 31, 34, 35, 37, 38, 41, 45, 46). Although this dietary pattern had been defined differently in different studies, in general, the traditional southern Chinese pattern was distinguished by substantial consumption of rice and vegetables, as well as moderate amounts of animal foods, especially pork. The forest plot in Figure 2A displays the risk of HTN in individuals with the highest category of this dietary pattern compared to the lowest category. A fixed-effects model was employed for data analysis, as no significant heterogeneity was observed (I^2^ = 10.7%, *p* = 0.329). The combined outcome demonstrated that greater adherence to traditional southern Chinese pattern was associated with a lower risk of HTN (OR = 0.95, 95% CI: 0.92–0.97, *p* < 0.001).

### 3.5 Fruit and dairy pattern and hypertension

Of the 24 studies, 14 identified the ‘fruit and dairy pattern’ as a dietary pattern; 2 of them were longitudinal studies (30, 47) and the remaining 12 were cross-sectional studies (27, 29, 31–33, 35–37, 41, 43, 45, 46). The fruit and dairy pattern tended to be highly loaded with fruit, milk and dairy products. A significantly reduced risk of HTN in the highest compared to the lowest category of the fruit and dairy pattern was apparent in Figure 2B (OR = 0.75, 95% CI: 0.64–0.89, *p* = 0.001), despite the considerable heterogeneity (I^2^ = 80.4%, *p* < 0.001). A random-effects model was implemented to integrate all investigations.

### 3.6 Animal food pattern and hypertension

Of the 24 studies, 14 identified the ‘animal food pattern’ as a dietary pattern; 2 of them were longitudinal studies (47, 48) and the remaining 12 were cross-sectional studies (27, 28, 35–42, 44). The animal food pattern was characterized to have high factor loadings for foods including pork and other livestock meat, poultry, animal organs, aquatic products/seafood, processed and cooked meat and eggs. The meta-analysis found no relationship between animal food pattern and HTN (OR = 1.06, 95% CI: 0.98, 1.15, *p* = 0.171) (Figure 2C). The random-effects model was employed to evaluate the data from these investigations due to the substantial heterogeneity (I^2^ = 51.9%, *p* = 0.007).

### 3.7 Subgroup analysis

A subgroup analysis was carried out in order to further explore the correlation among dietary patterns and HTN risk (Table 4). We found that dietary patterns and HTN were influenced by study design, sample size, and whether or not to adjust for multiple confounders. The negative association of the traditional southern Chinese pattern with HTN was evident in studies with cross-sectional designs, small sample sizes, and corrections for smoking, alcohol, physical activity, body mass index (BMI), or energy. The correlation between fruit and dairy pattern and HTN was more pronounced in studies with cross-sectional designs, small sample sizes, and corrections for smoking, alcohol, and BMI, but not for physical activity. In addition, we discovered a substantial positive correlation between animal food pattern and HTN in studies with a large sample size. As the impact of these variables is significantly in determining the linkage between dietary patterns and HTN risk, their disparities could potentially account for the observed heterogeneity across different studies.

**Table 4:** Subgroup analyses of dietary patterns and hypertension.

Dietary patterns	No. of studies	Combined risk estimate		Test of heterogeneity		
		OR (95% CI)	*P*	Q	I^2^%	*P*
Chinese traditional southern	17	0.95 (0.92,0.97)	< 0.001	17.92	10.7	0.329
Study design						
Cross-sectional	13	0.94 (0.91,0.97)	< 0.001	11.82	0.0	0.460
Cohort	4	1.01 (0.92,1.11)	0.832	3.93	23.6	0.270
Sample size						
Large (≥ 5,000)	4	0.97 (0.89,1.07)	0.573	1.72	0.0	0.633
Small (< 5,000)	13	0.94 (0.91,0.97)	< 0.001	15.73	23.7	0.204
Adjustment for confounders						
Smoking						
Yes	12	0.95 (0.92,0.97)	< 0.001	10.25	0.0	0.508
No	5	0.93 (0.78,1.10)	0.391	7.62	47.5	0.106
Alcohol						
Yes	11	0.94 (0.91,0.97)	< 0.001	13.83	27.7	0.181
No	6	1.05 (0.93,1.19)	0.404	0.95	0.0	0.967
Physical activity						
Yes	10	0.95 (0.92,0.97)	< 0.001	9.32	3.4	0.408
No	7	0.94 (0.80,1.09)	0.399	8.58	30.1	0.198
BMI						
Yes	10	0.94 (0.91,0.97)	< 0.001	11.62	22.5	0.236
No	7	1.06 (0.93,1.21)	0.396	3.36	0.0	0.762
Energy						
Yes	10	0.95 (0.92,0.97)	< 0.001	9.76	7.8	0.371
No	7	0.93 (0.80,1.08)	0.349	8.12	26.1	0.229
Fruit and dairy	15	0.75 (0.64,0.89)	0.001	71.51	80.4	< 0.001
Study design						
Cross-sectional	13	0.70 (0.57,0.86)	0.001	54.15	77.8	< 0.001
Cohort	2	/	/	/	/	/
Sample size						
Large (≥ 5,000)	3	0.99 (0.89,1.09)	0.797	2.48	19.4	0.289
Small (< 5,000)	12	0.67 (0.55,0.82)	< 0.001	44.02	75.0	< 0.001
Adjustment for confounders						
Smoking						
Yes	11	0.74 (0.61,0.90)	0.002	64.14	84.4	< 0.001
No	4	0.78 (0.56,1.10)	0.162	6.90	56.5	0.075
Alcohol						
Yes	9	0.69 (0.55,0.87)	0.002	59.19	86.5	< 0.001
No	6	0.87 (0.69,1.09)	0.218	10.26	51.3	0.068
Physical activity						
Yes	8	0.90 (0.80,1.02)	0.090	14.38	51.3	0.045
No	7	0.55 (0.38,0.80)	0.002	33.78	82.2	< 0.001
BMI						
Yes	9	0.71 (0.56,0.89)	0.003	57.66	86.1	< 0.001
No	6	0.81 (0.64,1.04)	0.098	13.23	62.2	0.021
Energy						
Yes	7	0.85 (0.72,1.00)	0.049	20.18	70.3	0.003
No	8	0.65 (0.47,0.90)	0.009	40.39	82.7	< 0.001
Animal food	17	1.06 (0.98,1.15)	0.171	33.26	51.9	0.007
Study design						
Cross-sectional	15	1.04 (0.95,1.15)	0.409	30.35	53.9	0.007
Cohort	2	/	/	/	/	/
Sample size						
Large (≥ 5,000)	6	1.12 (1.03,1.22)	0.012	11.18	55.3	0.048
Small (< 5,000)	11	0.98 (0.86,1.11)	0.751	15.62	36.0	0.111
Adjustment for confounders						
Smoking						
Yes	13	1.07 (0.98,1.17)	0.144	30.52	60.7	0.002
No	4	0.98 (0.81,1.19)	0.865	1.94	0.0	0.584
Alcohol						
Yes	12	1.06 (0.97,1.16)	0.193	29.88	63.2	0.002
No	5	1.02 (0.86,1.22)	0.808	3.11	0.0	0.540
Physical activity						
Yes	13	1.06 (0.98,1.15)	0.172	26.36	54.5	0.010
No	4	1.07 (0.77,1.47)	0.699	6.75	55.6	0.080
BMI						
Yes	13	1.07 (0.97,1.18)	0.198	28.58	58.0	0.005
No	4	1.00 (0.91,1.10)	0.994	2.02	0.0	0.569
Energy						
Yes	4	1.05 (0.91,1.20)	0.518	5.37	44.1	0.147
No	13	1.07 (0.96,1.19)	0.228	27.55	56.4	0.006

OR, odds ratio. CI, confidence interval. BMI, body mass index.

### 3.8 Sensitivity analysis

**Figure 3:**
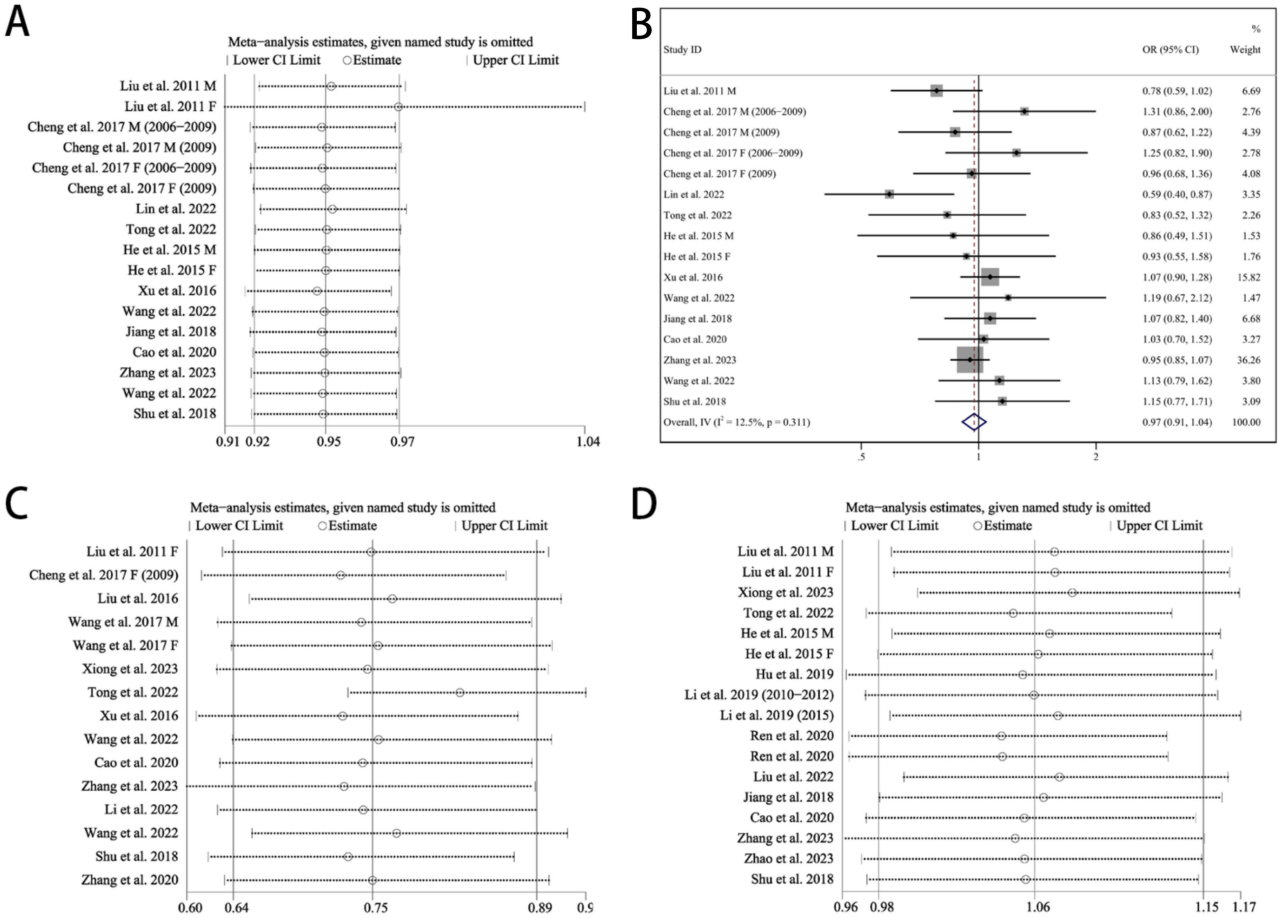
Sensitivity analysis. **(A)** Chinese traditional southern dietary pattern, **(B)** forest plots of Chinese traditional southern dietary pattern and hypertension after omitting the study of Liu (27) Female, **(C)** fruit and dairy dietary pattern, **(D)** animal food dietary pattern. OR, odds ratio. CI, confidence interval. M, male. F, female.

In the analysis of the traditional southern Chinese pattern, the results were unstable, which became statistically non-significant on the exclusion of the study of Liu (27) Female (OR = 0.97, 95% CI: 0.91–1.04) (Figures 3A,B). For the fruit and dairy pattern and animal food pattern, the overall statistical significance of the meta-analysis did not change after the deletion of any individual study, indicating that the results were stable and credible (Figures 3C,D).

### 3.9 Publication bias

**Figure 4:**
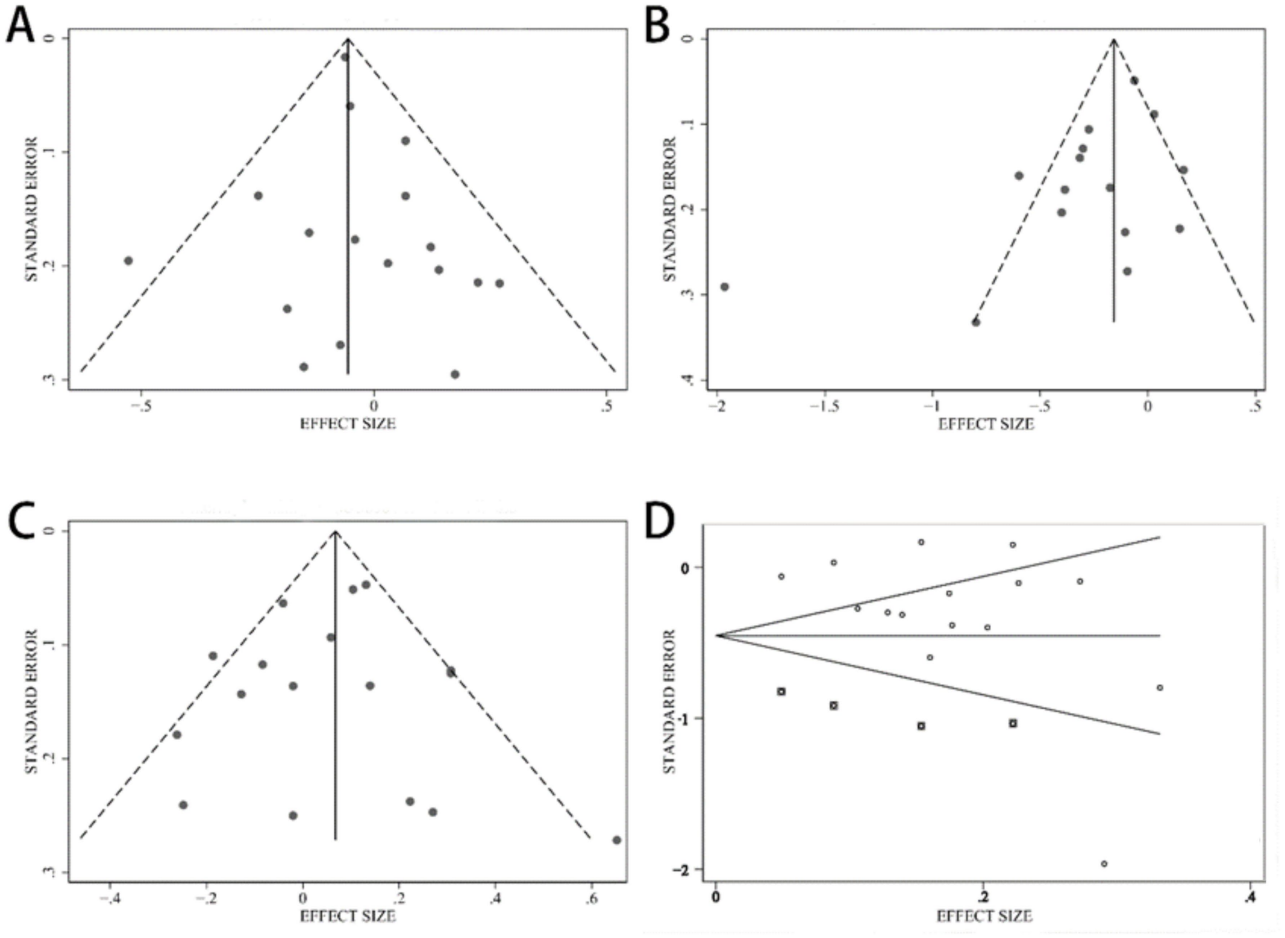
Funnel plot. **(A)** Chinese traditional southern dietary pattern, **(B)** fruit and dairy dietary pattern before using the trim-and-fill method, **(C)** animal food dietary pattern, **(D)** fruit and dairy dietary pattern after using the trim-and-fill method, and the boxes represent the filled studies.

No publication bias occurred in the traditional southern Chinese pattern studies (Begg’s test *p* = 0.484, Egger’s test *p* = 0.452) (Figure 4A) and animal food pattern studies (Begg’s test *p* = 0.837, Egger’s test *p* = 0.793) (Figure 4C) according to Begg’s and Egger’s tests. Despite the notable publication bias in the research on fruit and dairy pattern (Begg’s test *p* = 0.092, Egger’s test *p* = 0.046) (Figure 4B), the correlation continued to be significant following the inclusion of four more studies through the trim-and-fill method (OR = 0.64, 95% CI: 0.52–0.78, *p* < 0.001) (Figure 4D).

## 4 Discussion

As far as we know, this meta-analysis provides the most up-to-date findings on how dietary patterns impact the likelihood of developing HTN among Chinese adults. In the current study, the results indicated that the traditional southern Chinese pattern and the fruit and dairy pattern were relevant to a lower risk of HTN, whereas the animal food pattern had nothing to do with HTN risk. We incorporated data from 24 observational studies that involved 159,096 participants in this analysis.

Although different definitions are used, the traditional southern Chinese pattern consists mainly of grains (especially rice), vegetables, and meat (especially pork). It seems probable that the consumption of these foods is a significant contributor to the observed influence on HTN risk, especially vegetables. Vegetable consumption and HTN risk were discovered to be negatively correlated in a meta-analysis involving eight cohorts (49). Vegetables are a valuable source of dietary fiber, vitamins, minerals and phytochemicals (50). A systematic review conducted by Vitrag and his colleagues showed that a positive association was consistently observed between increased dietary fiber intake and lower BP levels (51). Dietary fiber affects blood pressure by forming a viscous gel in the gastrointestinal tract, delaying glucose absorption and digestion, and improving blood sugar control (52). Furthermore, the gut flora can ferment dietary fiber to synthesize short-chain fatty acids (SCFAs), which have the ability to reduce BP (53, 54). Relevant meta-analyses revealed that serum vitamin C concentration was oppositely related to both systolic blood pressure (SBP) and diastolic blood pressure (DBP) (55), and vitamin C supplementation could reduce SBP by −3.0 mmHg (95% CI, −4.7 to −1.3 mmHg; *p* = 0.001) over the short term (56). As an effective antioxidant and free radical scavenger, vitamin C can reduce the damage of oxidative stress to vascular endothelial cells (57–60). In addition, vitamin C potentiates the synthesis and bioactivity of endothelial nitric oxide (NO) (a vital antioxidant and vasodilator) by increasing intracellular tetrahydrobiopterin (61, 62). Vitamin E also reduces BP through a number of mechanisms and its main biological function is antioxidant (63–65). Vegetables are good sources of magnesium and potassium, whose role in the regulation of BP is well established in previous studies (66–68). The mechanisms of potassium’s hypotensive effect include: promoting sodium excretion in urine, endothelium-dependent vasodilation, increasing endothelial NO production, and inhibiting the sympathetic nervous system (69). It has been reported that magnesium ions can directly stimulate the production of prostacyclin and NO, induce vasodilation, act as a natural calcium channel blocker, decrease intracellular calcium and sodium, and thus reduce peripheral vascular resistance and BP (70). The flavonoids and carotenoids contained in vegetables regulate BP by their ability to inhibit free radicals, reduce reactive oxygen species-induced damage, reduce oxidative stress and inflammatory responses, and increase NO bioavailability and bioactivity (71, 72). A dose–response meta-analysis of prospective cohort studies showed that compared to non-flavonoid consumption, the risk of incident HTN was reduced by 3% (relative risk (RR): 0.97, 95% CI: 0.94–0.99) at 500 mg/d intake (73). A meta-analysis by Behzadi et al. (74) revealed that carotenoid supplementation prominently reduced SBP (weighted mean difference (WMD), −2.492 mmHg; 95% Cl, −4.52, −0.47; *p* = 0.016) and DBP (WMD, −1.60 mmHg; 95% Cl, −2.73, −0.47; *p* = 0.005). A meta-analysis carried out by Jiang and others (75) with 23 articles determined that traditional Chinese dietary patterns characterized were related to a lower risk of obesity, which was regarded as a crucial risk factor for HTN.

Compared to the lowest category of the fruit and dairy pattern, the highest category had a 25% reduction in HTN risk in our findings. This beneficial association can be explained by the beneficial influence of fruit and milk on BP. Recently, Madsen and his colleagues conducted a meta-analysis including 18 prospective studies and demonstrated that fruit intake was connected to a decrease HTN risk (RR: 0.91, 95% CI: 0.85–0.97) (76). Fruits are rich in potassium, magnesium, vitamin C, folate, flavonoids, and carotenoids, which are thought to reduce BP by enhancing endothelial function, modulating baroreflex sensitivity, promoting vasodilation, and boosting antioxidant activity (58, 72, 73, 77–79). Recent meta-analysis carried out by Chen and his co-workers, hinted that total dairy intake was linked to a diminished HTN risk (RR for highest compared with lowest level of intake: 0.91, 95% CI: 0.86–0.95; RR for 1 serving/d increase: 0.96, 95% CI: 0.94–0.97) (80). It has been proposed that the beneficial effects of milk and dairy products on HTN are attributed to a number of ingredients such as calcium, potassium, or lactate tripeptides (81–83). Dietary calcium can regulate BP by secreting parathyroid hormone, promoting sodium ion excretion, regulating the sympathetic nervous system, and changing intracellular free calcium levels (84). During milk fermentation, bioactive peptides are produced that inhibit angiotensin-converting enzyme, thereby maintaining normal BP (85).

Our findings showed no significant correlation between the animal food pattern and HTN. Our results contradicted some former studies that suggested a positive correlation between this dietary pattern and HTN risk (35, 39, 42, 47). This null relationship might have various reasonable causes. Above all, it may be due to interactions between different food categories. Numerous studies have confirmed that red meat consumption (especially processed red meat) and poultry consumption are related to an incremental risk of HTN, whereas the intake of fish or eggs is inversely connected with HTN risk, so the harmful effects may be offset by the protective effects of other food groups (86–89). Secondly, the intake of different parts of the same food was unknown, and the complex effects of various components after entering the body were unknown, which need to be further explored. For example, livestock meat is an excellent source of fat supply and a provider of high-quality protein (89). It has been reported that the protein hydrolysates of meat and muscle have strong vasopressor activity (90). A research completed by Zhou and others has proven that there is a window of consumption (at appropriate levels) of proteins, whether from fish, eggs, red meat sources (including unprocessed) or poultry sources, in which the risk of HTN is low (91). Thirdly, the zero correlation may lie in the fact that the sample size of the survey was not large enough. Subgroup analysis implied a significant positive correlation between HTN and the animal food pattern in studies with larger sample sizes. Compared to studies with large sample sizes, studies with small sample sizes have lower statistical validity and are more susceptible to extreme values or outliers affecting the overall results. More studies with larger sample sizes are needed to determine the true impact of the animal food pattern on the risk and severity of HTN. Finally, it pertained to a number of potential confounding factors that we did not take into consideration or that we were unable to quantify, such as cooking methods.

First of all, there was significant heterogeneity in the included studies. This could be attributed to extensive differences in the collection and analysis of dietary data, inconsistent adjustment of multifarious confounders, and the recognition of dietary patterns. While we endeavored to align the factor loads of included studies as closely as possible, the practical factor loads of the identical foods in semblable dietary patterns were never precisely consistent across studies. In subgroup analyses, we also observed that study design, sample size, adjustments for smoking, alcohol, physical activity, BMI, or energy had a significant impact on the dietary patterns and HTN. However, limited by the amount of research included, we were unable to investigate all possible sources of heterogeneity. Secondly, cross-sectional designs were employed in the majority of studies, which are more prone to recall and selection bias than cohort designs, particularly in case of diet recall bias. Furthermore, the cross-sectional studies were not suitable for causal reasoning and did not permit the evaluation of any potential trends. Thirdly, all studies included in this meta-analysis were conducted in China, and as dietary intake is influenced by ethnic, geographic, and social factors, the results cannot be extended to all populations. Last but not least, the instability of the results in sensitivity analysis of the traditional southern Chinese dietary pattern suggested that additional pertinent articles are required to delve into this connection. In the fruit and dairy pattern studies, publication bias was identified. Nonetheless, the inverse association remained significant after the pruning and filling method was used.

## 5 Conclusion

The results of our meta-analysis showed that Chinese traditional southern dietary pattern and fruit and dairy dietary pattern were strongly associated with the risk of HTN. Higher adherence to these two dietary patterns may be beneficial in reducing the risk of HTN. However, there was no evidence to indicate the level of risk of high, moderate or low risk of HTN based on dietary patterns. Subgroup analyses showed that the negative association between these two dietary patterns and the risk of HTN was influenced by study design, sample size, and multiple potential confounders. Future high-quality prospective studies with larger sample sizes and strict control of confounders are needed to further validate our findings and explore their dose–response relationships.

## Data Availability

The original contributions presented in the study are included in the article/supplementary material, further inquiries can be directed to the corresponding authors.
